# Supplementary material for: SS18 regulates pluripotent-somatic transition through phase separation
Source: Nat Commun. 2021 Jul 2;12:4090. doi: 10.1038/s41467-021-24373-5 (PMC8253816; doi:10.1038/s41467-021-24373-5)
Supplement: Supplementary file 1 — Supplementary Information [file 41467_2021_24373_MOESM1_ESM.pdf]

Supplemetary Figure 1

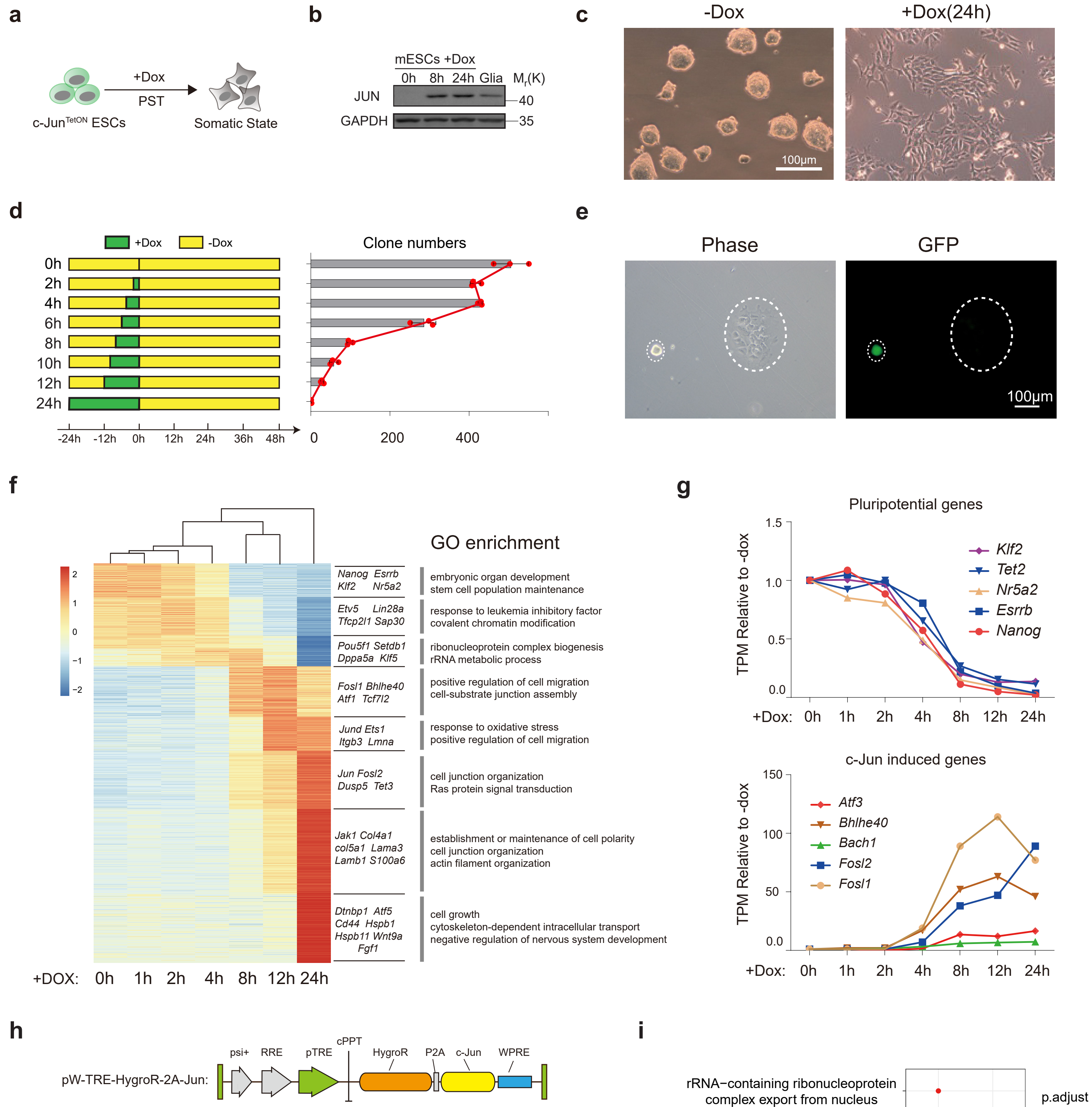

i

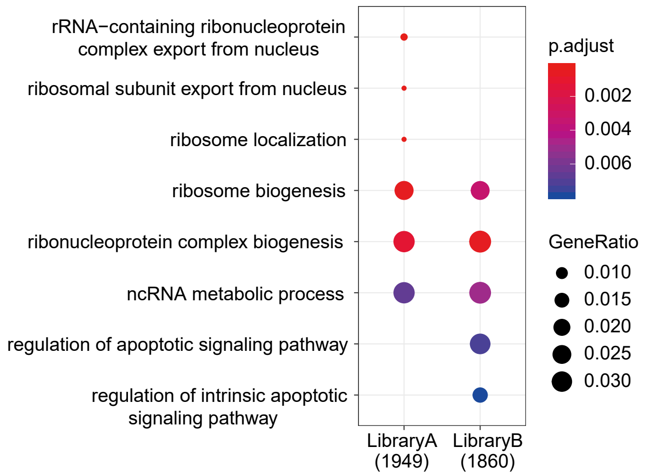

j

CRISPR/Cas9 Screening repeat analyzed by MAGeCK

Rank	Genes	p-value	fold change
1	**Jun**	2.20e-7	10.374
2	**Ss18**	2.20e-7	5.8864
3	Ric8	2.20e-7	9.0353
4	Smarca4	2.20e-7	8.9982
5	Trp53	6.60e-7	6.94
6	Rhoa	1.10e-6	8.2703
8	Myl6	4.18e-6	4.9431
10	Asf1a	1.50e-5	3.5994
12	Crampl	3.24e-5	5.9806
13	Myh9	3.63e-5	5.5341

Supplementary Figure 1

cJun induced pluripotent to somatic transition system and CRISPR/Cas9 screening system

(a) Schematic of cJUN induced pluripotent to somatic transition(PST). Dox, doxycycline.

(b) The JUN protein expression at 0hrs, 8hrs and 24hrs during PST was indicated by western blotting. The neonatal mice derived Glial cells with high expression of JUN were used as controls. Threes biological replicates.

(c) Images of c-Jun induced PST. Scale bars,100μm. Three biological replicates.

(d) The time requirement of c-Jun expression for c-Jun induced PST. Data are mean±s.d.; n = 3 independent experiments.

(e) Images of (d) in condition for dox induction 24h during c-Jun induced PST. The small white circles marked the clone that had not yet differentiated and the large circles marked the differentiated one. Scale bars,100μm. Three biological replicates.

(f) Heat maps and GO analysis for time course RNA-seq data by c-Jun induced PST in hour level resolution.

(g) The expression of represented pluripotent genes or first response genes in RNA-seq data.

(h) The construction of lentiviral TetON vector for HygroR-Jun expression, used in the CRISPR/Cas9 screening.

(i) Gene ontology analysis indicates the most significantly depleted gene sets comparing input 2 to input 1 in GeCKO screening process. One-sided hypergeometric test adjusted for multiple comparisons.

(j) The CRISPR/Cas9 screening repeated and analyzed by MAGeCK[43] algorithm. The top hits were similar with figure 1d and the top two were marked by red.

Supplemetary Figure 2

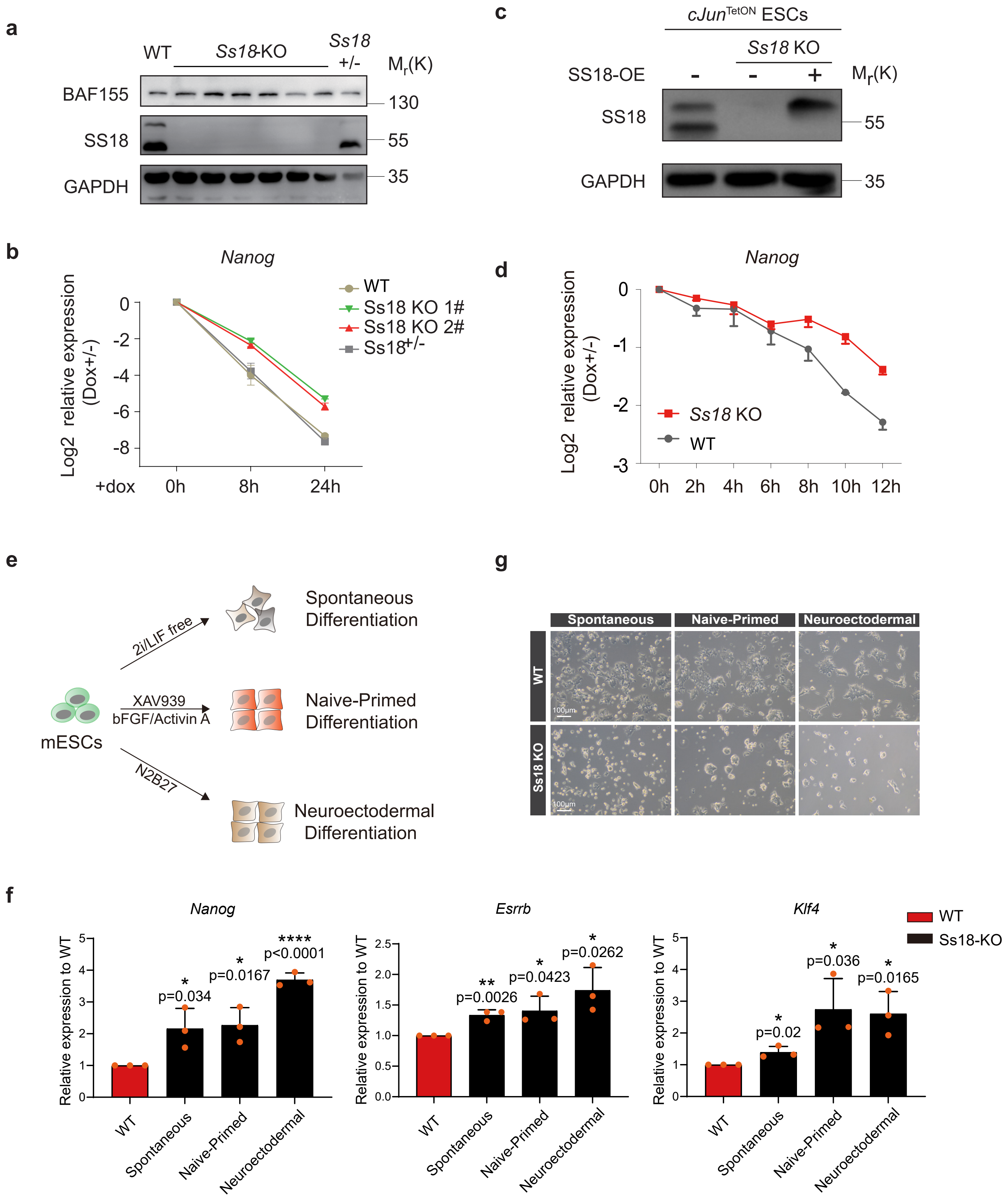

Supplementary Figure 2

SS18 mediates cJun induced PST

(a) The *Ss18*-Knockout ESCs were validated by western blotting. Three biological replicates.

(b) The expression of Nanog during c-Jun based PST at 8h and 24h for SS18 WT and KO ESCs. n = 2 biological replicates.

(c) Western blotting showed the overexpression of long isoform SS18 in *Ss18* deficiency Jun^{TetON} mESCs. Three biological replicates.

(d) Nanog relative expression when recover for 48h after induction of cJUN at indicated time series in SS18 WT and KO ESCs. n = 2 biological replicates.

(e) Schematic shows the commonly used differentiation systems. The details are in the method section.

(f) The relative expression of *Nanog*/*Esrrb*/*Klf4* of SS18 WT and KO mESCs after differentiation in (e). Data are mean±s.d., two-sided, unpaired t-test; n=3 independent experiments, * $p < 0.05$, ** $p < 0.01$, **** $p < 0.0001$.

(g) Representative images of SS18 WT and KO mESCs after differentiation in (e). Three biological replicates.

Supplemetary Figure 3

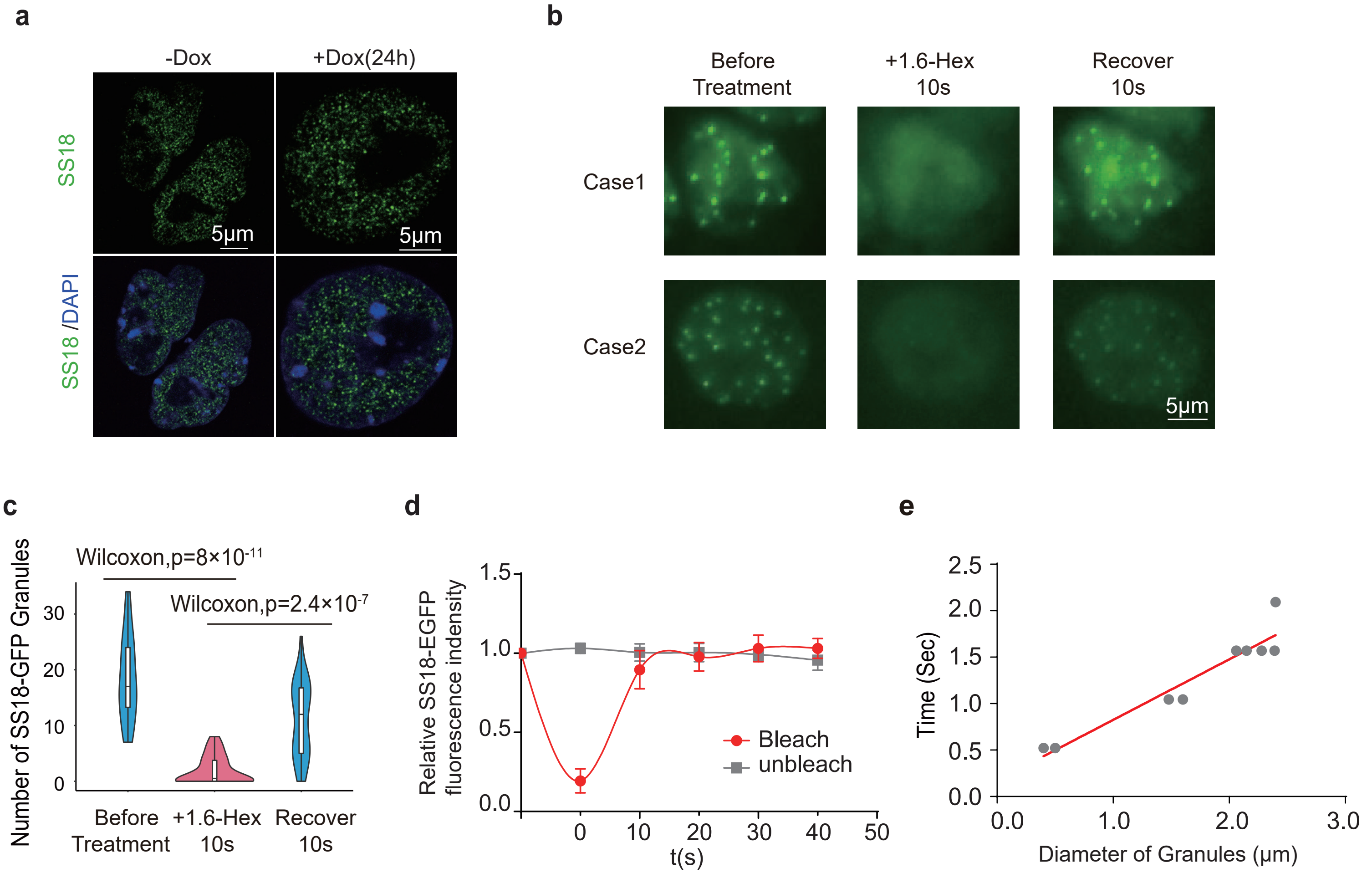

Supplementary Figure 3

Characteristics for the condensates formed by SS18

(a) Immunofluorescence imaging for SS18 in mESCs. Image processed by background subtraction. Scale bars, 5μm. laser intensity 2.5%, detector gain 500V, background subtraction treatment. Three biological replicates.

(b) Representative images of mESCs expressing SS18-GFP when treated with 3% 1.6-hexanediol (1.6-hex). Scale bars, 5μm. n = 3 biological replicates.

(c) The violin-plot counted the number of granules from 30 nucleus before and after 1.6-hex treatment. Two-sided Wilcoxon test adjusted for multiple comparisons. Before Treatment, Minimum 7, 25%Percentile 12.75, Median 17, 75%Percentile 24.25, Maximum 34; +1.6-Hex 10s, Minimum 0, 25%Percentile 0, Median 0.5, 75%Percentile 4, Maximum 8; Recover 10s, Minimum 0, 25%Percentile 5, Median 12, 75%Percentile 17.25, Maximum 26.

(d) Quantification of SS18-EGFP signaling in FRAP experiments. means ±SD. n = 7 condensates from 2 independent experiments.

(e) Time scale of granules fusion and fission events in cells with overexpression of SS18-EGFP by transfection, as a function of the diameter of granules. The time scale and granules size are approximately linearly positively correlated. The fitting slope K=0.65±0.19.

Supplemetary Figure 4

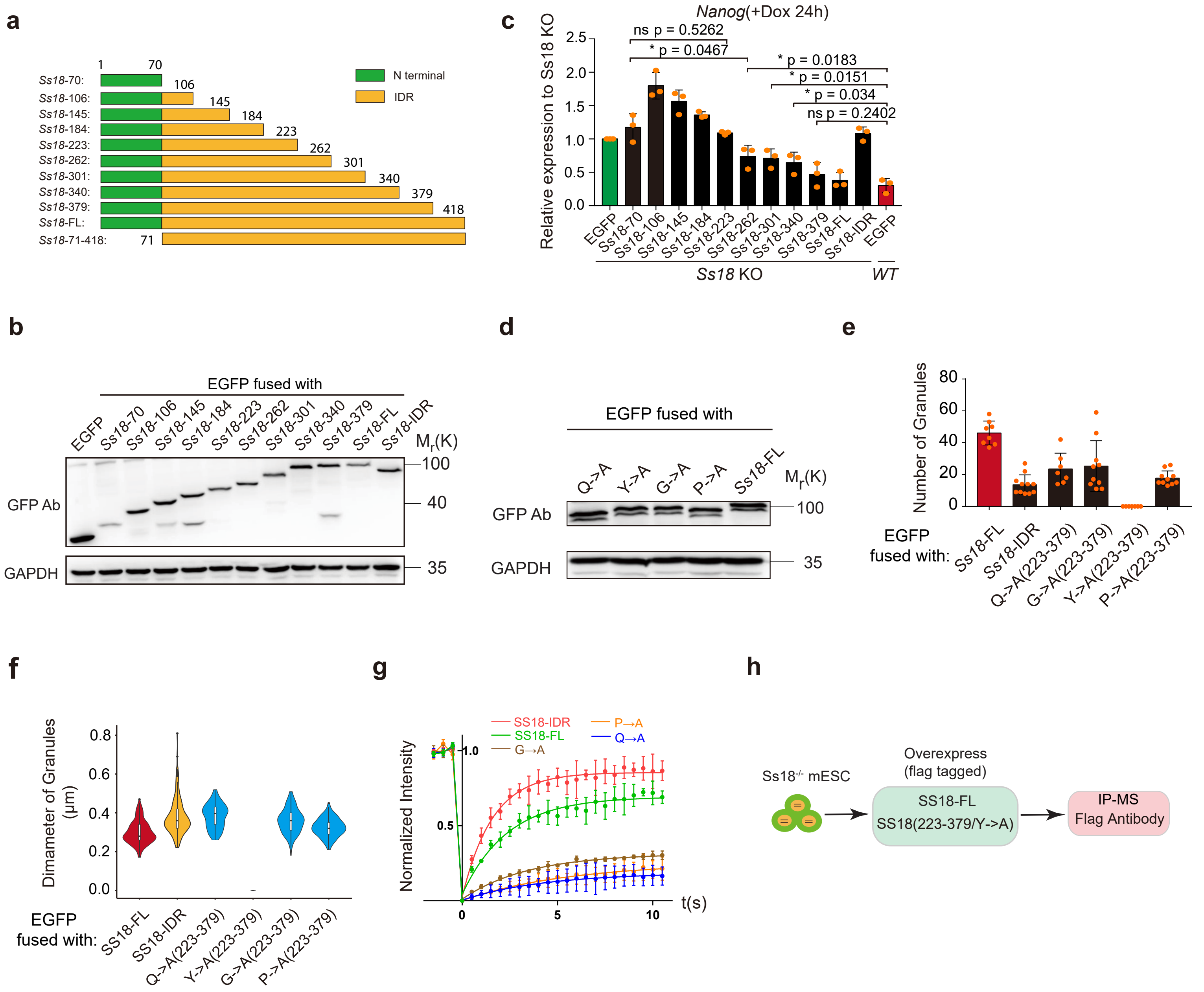

Supplementary Figure 4
Function analysis for SS18 IDR in JUN induced PST

(a) Schematic illustration of SS18 structure and truncation. Green color, N terminal; Orange, IDR.
(b) The expression of *Ss18* truncation mutants were confirmed by western blotting. n = 3 independent experiments.
(c) The expression of *Nanog* in *Ss18*-/- mESCs overexpressing the indicated *Ss18* truncation mutant by lentivirus during c-Jun based PST. Data are mean±s.d., two-sided, unpaired t-test; n=3 independent experiments, * $p < 0.05$.
(d) The expression of *Ss18* mutants were confirmed by western blotting. Three independent experiments.
(e) Histogram showed the number of granules within the mESCs expressing various *Ss18* mutants. Data are mean±s.d., Ss18-FL (n = 8 cells); Ss18-IDR (n = 11 cells); Q->A(223-379) (n = 7 cells); G->A(223-379) (n = 10 cells); Y->A(223-379) (n = 7 cells); P->A(223-379) (n = 10 cells);.
(f) The diameters of the granules of various SS18 mutants were indicated by violin plot. Ss18-FL (n=185 granules in 8 cells), Minimum 0.17, 25%Percentile 0.26, Median 0.28, 75%Percentile 0.34, Maximum 0.47; Ss18-IDR (n=129 granules in 11 cells), Minimum 0.22, 25%Percentile 0.32, Median 0.36, 75%Percentile 0.425, Maximum 0.81; Q->A (223-379) (n=88 granules in 7cells), Minimum 0.26, 25%Percentile 0.34, Median 0.395, 75%Percentile 0.43, Maximum 0.52; G->A (223-379) (n=146 granules in 10 cells), Minimum 0.18, 25%Percentile 0.31, Median 0.36, 75%Percentile 0.4, Maximum 0.51; Y->A (223-379) (n=7 cells); P->A (223-379) (n=105 granules in 10 cells), Minimum 0.21, 25%Percentile 0.29, Median 0.32, 75%Percentile 0.355, Maximum 0.45.
(g) The intensity dynamics inside and outside of the bleached region within granules (n=8) was fit to an exponential function. t=0 was the bleached time point. τ(SS18-IDR) =1.592s, τ(SS18-FL) =2.386s, τ(G-A) =3.496s, τ(P-A) =5.581s, τ(Q-A) =4.314s; Plateau (SS18-IDR) =85.45%, Plateau (SS18-FL) = 69.24%, Plateau(G-A) =31.42%, Plateau(P-A) =25.13%, Plateau(Q-A) =18.62%; diffusion coefficient of SS18-FL and SS18-IDR on the order of 5um^2/s, the three mutants on the order of 1um^2/s. Data are mean±s.d., n = 8 independent condensates.
(h) Schematic illustration of IP-Mass Spectrometry experiment in figure 4f. *Ss18* deficiency mESCs with overexpression of flag tagged SS18-FL, SS18(223-379/Y->A) by lentiviral infection were compared with *Ss18* deficiency mESCs with overexpression of EGFP.

Supplemetary Figure 5

a

	Length(aa)	Number of Tyr	Tyr ratio(%)
SS18-IDR	348	32	9.20
FUS-IDR	211	27	12.8
TAF15-IDR	177	26	14.9
BRD4-IDR	715	2	0.28
MED1-IDR	621	6	0.97
polyY	24	24	100

b

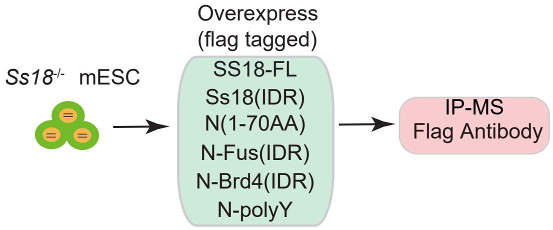

Supplementary Figure 5
Replacement of SS18 IDR

(a) The table shows the content of tyrosine within various fused IDR of Figure 5b.
(b) Schematic illustration of IP-Mass Spectrometry experiment in figure 5c. *Ss18* deficiency mESCs with overexpression of flag tagged SS18-FL, SS18(IDR), N terminal of SS18 or that fused with tyrosine polymer or IDR from FUS or BRD4 by lentiviral infection were compared with *Ss18* deficiency mESCs with overexpression of EGFP.

Supplemetary Figure 6

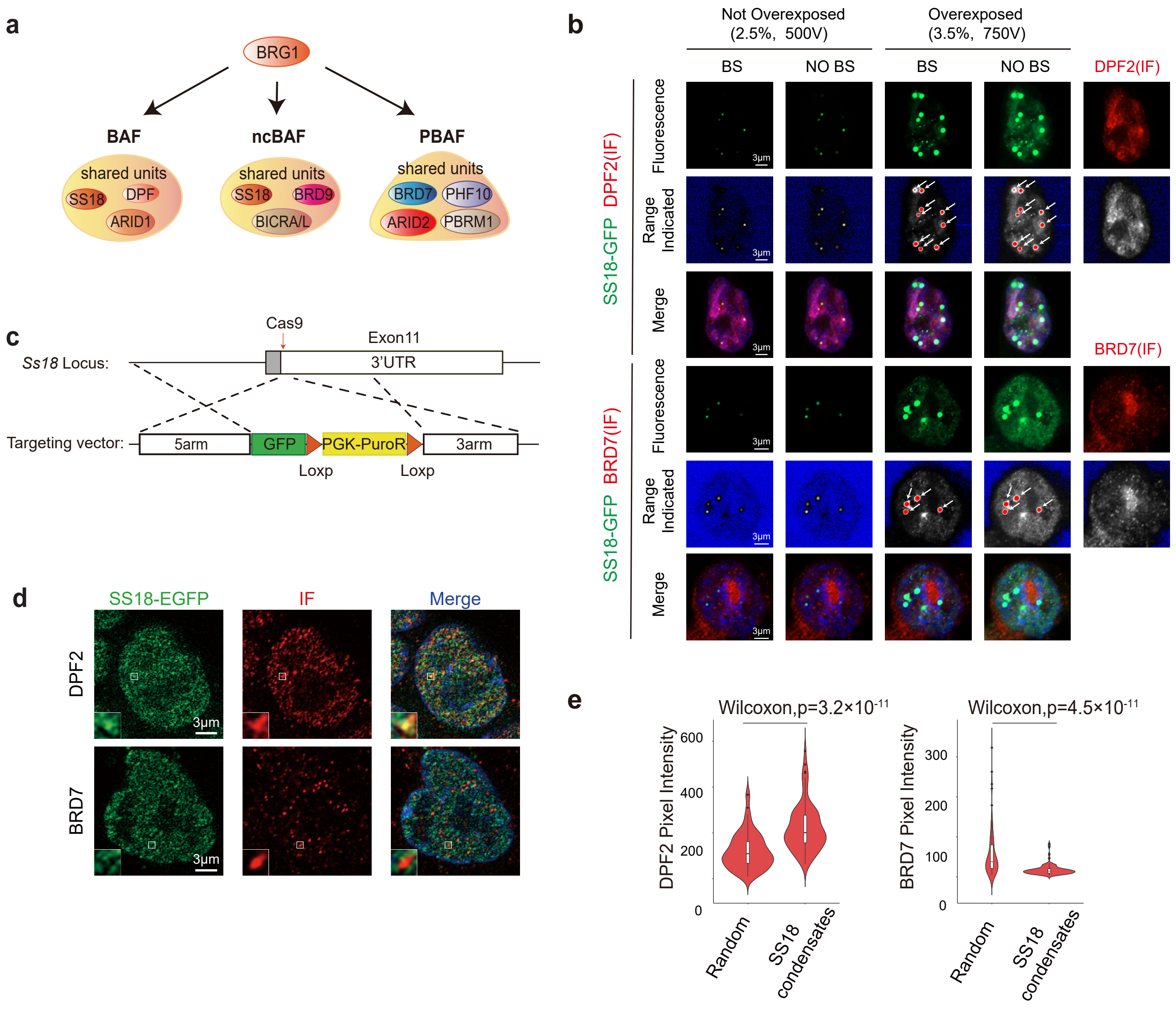

Supplementary Figure 6

SS18 condensates are associated exclusively with BAF, not PBAF.

(a) Schematic illustration shows that BRG1 is embedded in three subtypes of BAF complexes, i.e., BAF ncBAF and PBAF, whose specific subunits are displayed in the ovals.

(b) The comparison of different imaging processing. low laser intensity (2.5%) and detector gain (500V), with or without Background Subtraction (BS) treatment to avoid overexposure (left), compared with overexposed parameters (laser intensity 3.5%, detector gain 750V, middle). The parameters of photography of Immunofluorescence for DPF2 and BRD7 (right) were also laser intensity 3.5%, detector gain 750V. The overexposed pixels were marker by white arrows. Three biological replicates.

(c) Construct design for endogenous SS18-EGFP in mESCs. The cleavage site of Cas9 is close to stop codon of *Ss18* locus. The gray box shows the CDS region on the last exon.

(d) Representative images of endogenous SS18-EGFP and immunofluorescence for endogenous BAF specific subunit DPF2 (upper) and PBAF specific subunit BRD7 (lower). Image processed by background subtraction. Scale bars, 3μm. laser intensity 3.5%, detector gain 750V. Three biological replicates.

(e) Violin plot shows the co-localization analysis of (d). Random groups indicate the distribution of random pixels' fluorescence intensity of DPF2 and BRD7 within nuclei, respectively. SS18 condensates groups indicate the distribution of fluorescence intensity of DPF2 and BRD7 within random pixels of endogenous SS18-EGFP condensates. Two-sided Wilcoxon test adjusted for multiple comparisons. n = 80 pixels, from 5 nuclei. respectively. Random in left panel, Minimum 8.419, 25%Percentile 66.64, Median 109.3, 75%Percentile 163.7, Maximum 367.2; SS18 condensates in left panel, Minimum 65.14, 25%Percentile 153.2, Median 201, 75%Percentile 280.5, Maximum 557.3; Random in right panel, Minimum 4.603, 25%Percentile 20.58, Median 37.81, 75%Percentile 81.07, Maximum 323.1; SS18 condensates in right panel, Minimum 3.339, 25%Percentile 9.456, Median 14.36, 75%Percentile 20.35, Maximum 82.08.

Supplemetary Figure 7

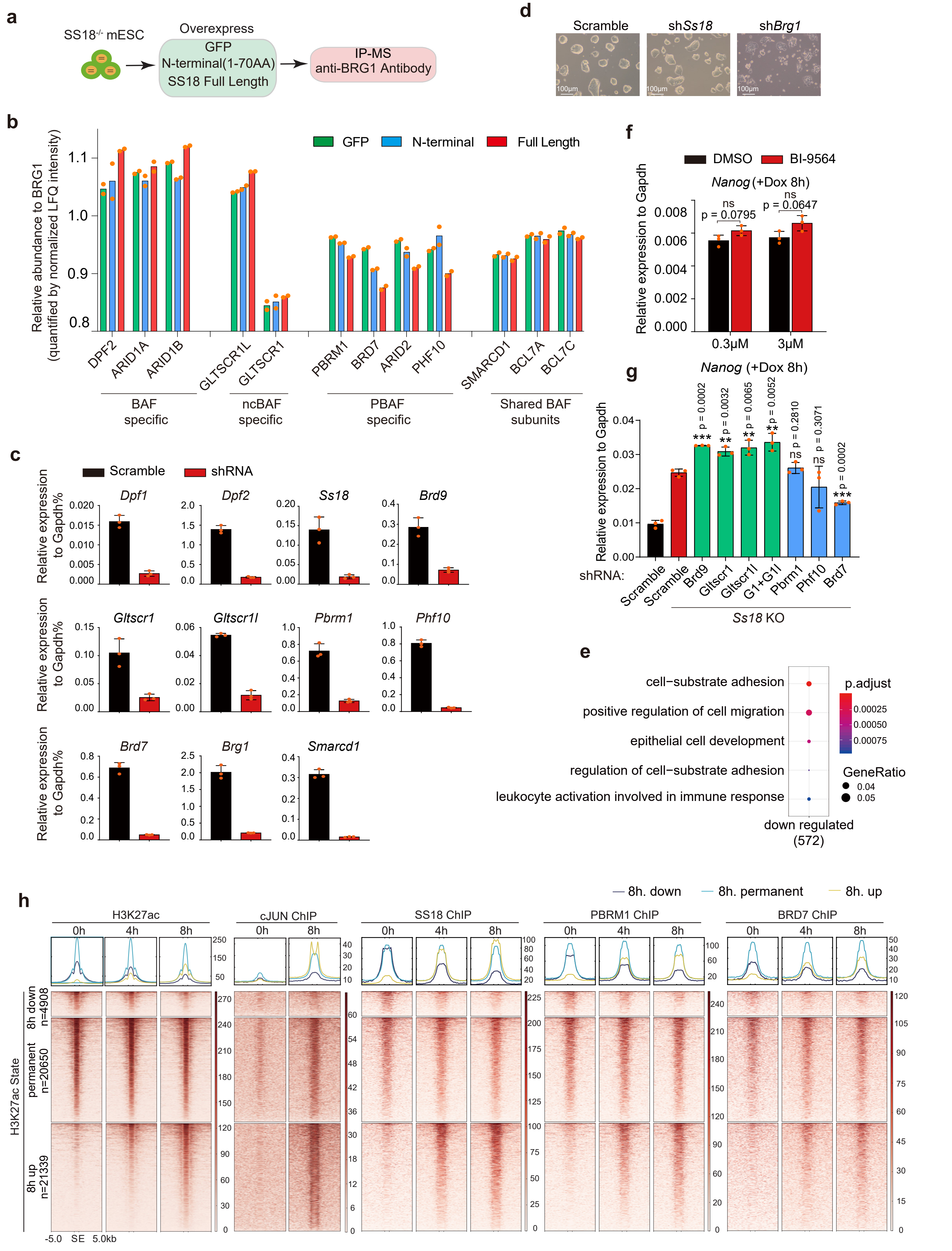

Supplementary Figure 7

Diverse subtypes of BAFs complex in PST.

(a) Schematic illustration of IP-Mass Spectrometry experiment in supplementary figure 7b. *Ss18* deficiency mESCs with overexpression of GFP, N-terminal and full length of SS18 by lentiviral infection were immunoprecipitated by anti-BRG1 antibody, followed by mass spectrometric detection.

(b) The histogram shows the relative precipitated abundance of indicated proteins to BRG1 as quantified by normalized LFQ intensity from IP-MS experiments by anti-BRG1 antibody performed on SS18KO mESCs expressing GFP, N-terminal (1-70AA) or SS18 as detailed in the supplementary figure 7a. IP-MS experiments were performed in duplicates and a two-sample t-test was applied. Two independent experiments.

(c) The knockdown efficiency related to Figure 6d. Data are mean±s.d., n = 3 independent experiments.

(d) Images of mESCs upon the knockdown of *Ss18* and *Brg1*. Scale bars,100μm. Three biological replicates.

(e) Gene ontology analysis indicated the most significantly down-regulated genes sets when knocking down *Brg1*. One-sided hypergeometric test adjusted for multiple comparisons.

(f) The expression levels of Nanog in Jun-FlagTetON mESCs at 8hrs during PST with or without treatment by BRD9 inhibitor, BI-9564. Data are mean±s.d., two-sided, unpaired t-test; n=3 independent experiments.

(g) The expression levels of Nanog in cJun-FlagTetON mESCs of SS18 deficiency at 8hrs during PST with knockdown of specific components of ncBAF and PBAF. G1+G1l, knockdown *Gltscr1* and *Gltscr1l* jointly. The p value indicates the significance between the marked sample and the SS18 KO control (the second scramble sample). Data are mean±s.d., two-sided, unpaired t-test; n=3 independent experiments, ** $p < 0.01$, *** $p < 0.001$.

(h) Heatmap showing the combination analysis for the ChIP-seq data of H3K27ac, cJUN, SS18, and PBAF specific subunits PBRM1 and BRD7. All the H3K27ac occupancy sites ± 5 kb of 0hs and 8hrs were divided into three categories (8hrs down, permanent and 8hrs up) according to the dynamic changes of intensity (fold change >2).

Original uncropped western blots

Supplementary Figure 1b

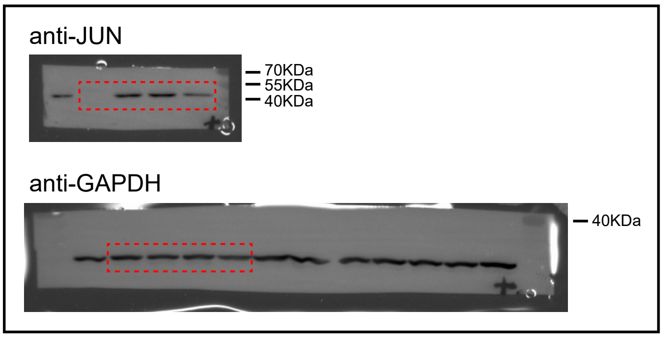

Supplementary Figure 2a

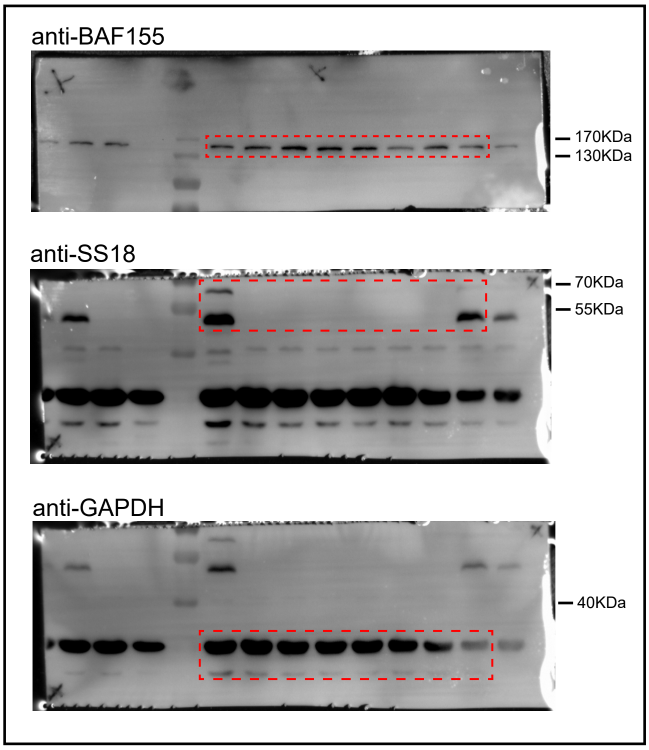

Supplementary Figure 2c

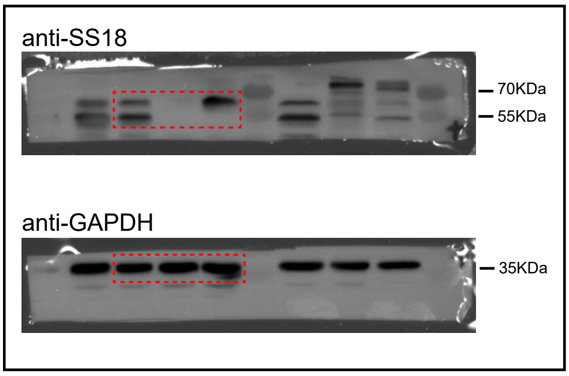

Supplementary Figure 4b

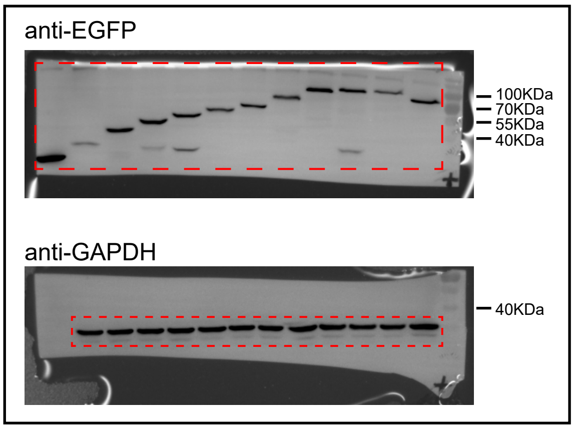

Supplementary Figure 4d

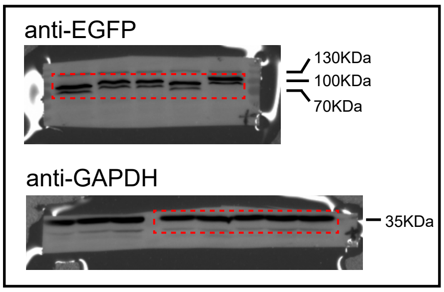

Supplementary Figure 8 Uncropped western blot images
