# Supplementary material for: SS18 regulates pluripotent-somatic transition through phase separation
Source: Nat Commun. 2021 Jul 2;12:4090. doi: 10.1038/s41467-021-24373-5 (PMC8253816; doi:10.1038/s41467-021-24373-5)
Supplement: Supplementary file 3 — Reporting Summary [file 41467_2021_24373_MOESM3_ESM.pdf]

nature research

Corresponding author(s): DBPR NCOMMS-21-03029A

Last updated by author(s): May 31, 2021

Reporting Summary

Nature Research wishes to improve the reproducibility of the work that we publish. This form provides structure for consistency and transparency in reporting. For further information on Nature Research policies, see Authors & Referees and the Editorial Policy Checklist .

Statistics

For all statistical analyses, confirm that the following items are present in the figure legend, table legend, main text, or Methods section.

n/a	Confirmed	
☐	☒	The exact sample size (*n*) for each experimental group/condition, given as a discrete number and unit of measurement
☐	☒	A statement on whether measurements were taken from distinct samples or whether the same sample was measured repeatedly
☐	☒	The statistical test(s) used AND whether they are one- or two-sided<br>*Only common tests should be described solely by name; describe more complex techniques in the Methods section.*
☒	☐	A description of all covariates tested
☒	☐	A description of any assumptions or corrections, such as tests of normality and adjustment for multiple comparisons
☐	☒	A full description of the statistical parameters including central tendency (e.g. means) or other basic estimates (e.g. regression coefficient) AND variation (e.g. standard deviation) or associated estimates of uncertainty (e.g. confidence intervals)
☐	☒	For null hypothesis testing, the test statistic (e.g. *F*, *t*, *r*) with confidence intervals, effect sizes, degrees of freedom and *P* value noted<br>*Give P values as exact values whenever suitable.*
☒	☐	For Bayesian analysis, information on the choice of priors and Markov chain Monte Carlo settings
☒	☐	For hierarchical and complex designs, identification of the appropriate level for tests and full reporting of outcomes
☒	☐	Estimates of effect sizes (e.g. Cohen's *d*, Pearson's *r*), indicating how they were calculated

Our web collection on statistics for biologists contains articles on many of the points above.

Software and code

Policy information about availability of computer code

Data collection	RNA-seq data and ChIP-seq data are collected at home by using the specific commercial kit(Illumina, NR601,MS-102-2001).
Data analysis	ChIP-seq data are mapped to the mm10 mouse genome assembly using bowtie2, version 2.4.1;Differential genes analysis, DEseq2, version 1.28.1; GO analysis: GO.db, version 3.11.4; clustering, Mfuzz, version 2.50.0 ; p value calculation, Prism 6; Mass spectrometry data analysis, MaxQuant version 1.6.0.1; FACS analysis, FlowJo 7.6.1; CRISPR/Cas9 screen data analysis, MAGeCK package5

For manuscripts utilizing custom algorithms or software that are central to the research but not yet described in published literature, software must be made available to editors/reviewers. We strongly encourage code deposition in a community repository (e.g. GitHub). See the Nature Research guidelines for submitting code & software for further information.

Data

Policy information about availability of data

All manuscripts must include a data availability statement. This statement should provide the following information, where applicable:

- Accession codes, unique identifiers, or web links for publicly available datasets
- A list of figures that have associated raw data
- A description of any restrictions on data availability

The RNA-Seq, ChIP-seq data have been deposited in the Gene Expression Omnibus database under the accession code GSE135451 [https://www.ncbi.nlm.nih.gov/geo/query/acc.cgi?acc=GSE135451]. The mass spectrometry proteomics data have been deposited to the ProteomeXchange Consortium (http://proteomecentral.proteomexchange.org) via the iProX partner repository with the dataset identifier PXD026208 [http://proteomecentral.proteomexchange.org/cgi/GetDataset?ID=PXD026208]. Source data are provided with this paper. The source data underlying Figures 1e, 2d, 2f, 3c,3d,4c, 5b, 6c and 6d and Supplementary Figures 1d, 1g, 2b, 2d, 2f, 3c-e,4c, 4e-g, 6e, 7b, 7c, 7f and 7g are provided as a Source Data file. Supplementary Data 1 and 2 are primers used in this study. The Cas9 screen data and IP-MS data are provided in Supplementary Data 3 and 4, respectively. The authors declare that all data supporting the findings of this study are available within the article and its supplementary information files or from the corresponding author upon reasonable request.

Field-specific reporting

Please select the one below that is the best fit for your research. If you are not sure, read the appropriate sections before making your selection.

☒ Life sciences ☐ Behavioural & social sciences ☐ Ecological, evolutionary & environmental sciences

For a reference copy of the document with all sections, see nature.com/documents/nr-reporting-summary-flat.pdf

Life sciences study design

All studies must disclose on these points even when the disclosure is negative.

Sample size	No statistical methods were used to determine sample size. The sample size we chosed is based on literatures in the field.
Data exclusions	No data were exclude from the analysis.
Replication	Data are presented as mean±s.d. as indicated in the Fig legends. For all the main figures from which the significance were assessed, at least 2 biological replication experiment were preformed, all attempts at replication were successful.
Randomization	In this study, we analyzed clone formation assay, qRT-PCR data, Western blot data, IP-MS data, RNA-seq data, ChIP-seq data with cell populations. We analyzed immuno-staining data with three random selected pictures.
Blinding	The investigates were blinded to group allocation during data collection and analysis.

Behavioural & social sciences study design

All studies must disclose on these points even when the disclosure is negative.

Study description	*Briefly describe the study type including whether data are quantitative, qualitative, or mixed-methods (e.g. qualitative cross-sectional, quantitative experimental, mixed-methods case study).*
Research sample	*State the research sample (e.g. Harvard university undergraduates, villagers in rural India) and provide relevant demographic information (e.g. age, sex) and indicate whether the sample is representative. Provide a rationale for the study sample chosen. For studies involving existing datasets, please describe the dataset and source.*
Sampling strategy	*Describe the sampling procedure (e.g. random, snowball, stratified, convenience). Describe the statistical methods that were used to predetermine sample size OR if no sample-size calculation was performed, describe how sample sizes were chosen and provide a rationale for why these sample sizes are sufficient. For qualitative data, please indicate whether data saturation was considered, and what criteria were used to decide that no further sampling was needed.*
Data collection	*Provide details about the data collection procedure, including the instruments or devices used to record the data (e.g. pen and paper, computer, eye tracker, video or audio equipment) whether anyone was present besides the participant(s) and the researcher, and whether the researcher was blind to experimental condition and/or the study hypothesis during data collection.*
Timing	*Indicate the start and stop dates of data collection. If there is a gap between collection periods, state the dates for each sample cohort.*
Data exclusions	*If no data were excluded from the analyses, state so OR if data were excluded, provide the exact number of exclusions and the rationale behind them, indicating whether exclusion criteria were pre-established.*
Non-participation	*State how many participants dropped out/declined participation and the reason(s) given OR provide response rate OR state that no participants dropped out/declined participation.*
Randomization	*If participants were not allocated into experimental groups, state so OR describe how participants were allocated to groups, and if allocation was not random, describe how covariates were controlled.*

Ecological, evolutionary & environmental sciences study design

All studies must disclose on these points even when the disclosure is negative.

Study description	*Briefly describe the study. For quantitative data include treatment factors and interactions, design structure (e.g. factorial, nested, hierarchical), nature and number of experimental units and replicates.*

Research sample	*Describe the research sample (e.g. a group of tagged Passer domesticus, all Stenocereus thurberi within Organ Pipe Cactus National Monument), and provide a rationale for the sample choice. When relevant, describe the organism taxa, source, sex, age range and any manipulations. State what population the sample is meant to represent when applicable. For studies involving existing datasets, describe the data and its source.*
Sampling strategy	*Note the sampling procedure. Describe the statistical methods that were used to predetermine sample size OR if no sample-size calculation was performed, describe how sample sizes were chosen and provide a rationale for why these sample sizes are sufficient.*
Data collection	*Describe the data collection procedure, including who recorded the data and how.*
Timing and spatial scale	*Indicate the start and stop dates of data collection, noting the frequency and periodicity of sampling and providing a rationale for these choices. If there is a gap between collection periods, state the dates for each sample cohort. Specify the spatial scale from which the data are taken*
Data exclusions	*If no data were excluded from the analyses, state so OR if data were excluded, describe the exclusions and the rationale behind them, indicating whether exclusion criteria were pre-established.*
Reproducibility	*Describe the measures taken to verify the reproducibility of experimental findings. For each experiment, note whether any attempts to repeat the experiment failed OR state that all attempts to repeat the experiment were successful.*
Randomization	*Describe how samples/organisms/participants were allocated into groups. If allocation was not random, describe how covariates were controlled. If this is not relevant to your study, explain why.*
Blinding	*Describe the extent of blinding used during data acquisition and analysis. If blinding was not possible, describe why OR explain why blinding was not relevant to your study.*

Did the study involve field work? ☐ Yes ☐ No

Field work, collection and transport

Field conditions	*Describe the study conditions for field work, providing relevant parameters (e.g. temperature, rainfall).*
Location	*State the location of the sampling or experiment, providing relevant parameters (e.g. latitude and longitude, elevation, water depth).*
Access and import/export	*Describe the efforts you have made to access habitats and to collect and import/export your samples in a responsible manner and in compliance with local, national and international laws, noting any permits that were obtained (give the name of the issuing authority, the date of issue, and any identifying information).*
Disturbance	*Describe any disturbance caused by the study and how it was minimized.*

Reporting for specific materials, systems and methods

We require information from authors about some types of materials, experimental systems and methods used in many studies. Here, indicate whether each material, system or method listed is relevant to your study. If you are not sure if a list item applies to your research, read the appropriate section before selecting a response.

Materials & experimental systems

n/a	Involved in the study	
☐	☒	Antibodies
☐	☒	Eukaryotic cell lines
☒	☐	Palaeontology
☐	☒	Animals and other organisms
☒	☐	Human research participants
☒	☐	Clinical data

Methods

n/a	Involved in the study	
☐	☒	ChIP-seq
☐	☒	Flow cytometry
☒	☐	MRI-based neuroimaging

Antibodies

Antibodies used	anti-SS18, CST, 21792; anti-JUN, abcam, ab31419; anti-GAPDH, KangChen Bio-tech, KC-5G5; anti-IgG, abcam, ab37415; anti-DPF2, Proteintech, 12111-1-AP-50μl; anti-BRD7, Proteintech, 51009-2-AP-50μl; anti-BRG1, CST, 52251; anti-PBRM1, Bethyl, A301-591A; anti-GFP, CST, 2555, anti-BAF155, Santa Cruz Biotechnology, sc-32763; anti-H3K27ac, abcam, ab4729.
Validation	Rabbit anti-SS18, CST, 21792, western blotting(WB) immunofluorescence(IF) and ChIP validation, https://www.cellsignal.cn/products/primary-antibodies/ss18-d6i4z-rabbit-mab/21792?site-search type=Products&N=4294956287&Ntt=ss18&fromPage=plp<br><br>Rabbit anti-JUN, abcam, ab31419, WB ChIP validation, https://www.abcam.cn/c-jun-antibody-ab31419.html

Mouse anti-GAPDH, KangChen Bio-Tech, KC-5G5, WB validation, http://www.aksomics.com/products/westernblot-internal-reference-1.html

Rabbit anti-DPF2 Proteintech, 12111-1-AP, IF validation, https://www.ptgcn.com/products/DPF2-Antibody-12111-1-AP.htm

Rabbit anti-BRD7 Proteintech, 51009-2-AP, IF and ChIP validation, https://www.ptgcn.com/products/BRD7-Antibody-51009-2-AP.htm

Mouse anti-BRG1 CST, 52251, IF and IP validation, https://www.cellsignal.cn/products/primary-antibodies/brg1-e9o6e-mouse-mab/52251?site-search-type=Products&N=4294956287&Ntt=brg1&fromPage=plp

Rabbit anti-PBRM1 bethyl, A301-591A, ChIP validation, https://www.bethyl.com/product/A301-591A/PBRM1+Antibody

Rabbit anti-GFP CST, 2555, WB validation, https://www.cellsignal.cn/products/primary-antibodies/gfp-antibody/2555?site-search-type=Products&N=4294956287&Ntt=gfp&fromPage=plp

Mouse anti-BAF155 Santa Cruz Biotechnology, sc-32763, WB validation, https://www.scbt.com/p/baf155-antibody-dxd7

Rabbit anti-H3K27ac abcam, ab4729, ChIP validation, https://www.abcam.cn/histone-h3-acetyl-k27-antibody-chip-grade-ab4729.html

Eukaryotic cell lines

Policy information about cell lines

Cell line source(s)

OG2ES were derived from E3.5 mouse blastocysts generated by mating homozygous Oct4-GFP transgenic allele-carrying mice (CBA/CaJ X C57BL/6J) with 129/Sv female mice

HEK293T was obtained from ATCC (CRL-1126)

Authentication

All ESC cell lines were characteristic in home by chimeric mice test.
HEK293T has no authentication

Mycoplasma contamination

All of the cell lines have been confirmed as mycoplasma contamination free with the kit from lonza(LT07-318)

Commonly misidentified lines
(See ICLAC register)

No

Palaeontology

Specimen provenance

Provide provenance information for specimens and describe permits that were obtained for the work (including the name of the issuing authority, the date of issue, and any identifying information).

Specimen deposition

Indicate where the specimens have been deposited to permit free access by other researchers.

Dating methods

If new dates are provided, describe how they were obtained (e.g. collection, storage, sample pretreatment and measurement), where they were obtained (i.e. lab name), the calibration program and the protocol for quality assurance OR state that no new dates are provided.

☐ Tick this box to confirm that the raw and calibrated dates are available in the paper or in Supplementary Information.

Animals and other organisms

Policy information about studies involving animals; ARRIVE guidelines recommended for reporting animal research

Laboratory animals

Oct-GFP trans genetic allele carrying male mice(male CBA/cAJ x female C57bl/6J) , female 129/Sv mice.
The mice used in this study are all one month old.
The housing conditions for mice: 12hrs light/12hrs dark, 25 degrees Celsius, 50% relative humidity.‫خ‬

Wild animals

No

Field-collected samples

No

Ethics oversight

All the animal experiments were performed with the approval and according to the guidelines of the animal care and use committee of the Guangzhou Institutes of Biomedicine and Health.

Note that full information on the approval of the study protocol must also be provided in the manuscript.

Human research participants

Policy information about studies involving human research participants

Population characteristics	NO
Recruitment	NO
Ethics oversight	NO

Note that full information on the approval of the study protocol must also be provided in the manuscript.

Clinical data

Policy information about clinical studies

All manuscripts should comply with the ICMJEguidelines for publication of clinical research and a completedCONSORT checklist must be included with all submissions.

Clinical trial registration	NO
Study protocol	NO
Data collection	NO
Outcomes	NO

ChIP-seq

Data deposition

- [x] Confirm that both raw and final processed data have been deposited in a public database such as GEO.
- [x] Confirm that you have deposited or provided access to graph files (e.g. BED files) for the called peaks.

Data access links
May remain private before publication.

The accession number for the RNA-seq and ChIP-Seq data in this paper is GSE135451.

Files in database submission

1) raw data
H3K27ac-ChIP-seq.0h.fq.gz
H3K27ac-ChIP-seq.4h.fq.gz
H3K27ac-ChIP-seq.8h.fq.gz
cJUN-ChIP-seq.8h.fq.gz
SS18-ChIP-seq.0h.fq.gz
SS18-ChIP-seq.4h.fq.gz
SS18-ChIP-seq.8h.fq.gz
PBRM1-ChIP-seq.0h.fq.gz
PBRM1-ChIP-seq.4h.fq.gz
PBRM1-ChIP-seq.8h.fq.gz
BRD7-ChIP-seq.0h.fq.gz
BRD7-ChIP-seq.4h.fq.gz
BRD7-ChIP-seq.8h.fq.gz

2) processed data
H3K27ac-ChIP-seq.0h.bw
H3K27ac-ChIP-seq.4h.bw
H3K27ac-ChIP-seq.8h.bw
cJUN-ChIP-seq.8h.bw
SS18-ChIP-seq.0h.bw
SS18-ChIP-seq.4h.bw
SS18-ChIP-seq.8h.bw
PBRM1-ChIP-seq.0h.bw
PBRM1-ChIP-seq.4h.bw
PBRM1-ChIP-seq.8h.bw
BRD7-ChIP-seq.0h.bw
BRD7-ChIP-seq.4h.bw
BRD7-ChIP-seq.8h.bw

Genome browser session (e.g. UCSC)

no longer applicable

Methodology

Replicates

ChIP-seq experiments were performed in cJUN-TetON mESCs treated by Dox for 0hrs, 4hrs and 8hrs with one time.

Sequencing depth

H3K27ac-ChIP-seq.0h.fq.gz 3,402,055 2,260,170 75bp single-end
H3K27ac-ChIP-seq.4h.fq.gz 4,789,284 3,528,504 75bp single-end
H3K27ac-ChIP-seq.8h.fq.gz 6,937,612 5,077,507 75bp single-end
Jun-ChIP-seq.8h.fq.gz 10,905,581 6,333,485 75bp single-end
SS18-ChIP-seq.0h.fq.gz 16363079 12998286 150bp single-end
SS18-ChIP-seq.4h.fq.gz 24729444 20468065 150bp single-end
SS18-ChIP-seq.8h.fq.gz 24062762 20332973 150bp single-end
PBRM1-ChIP-seq.0h.fq.gz 22870777 16827542 150bp single-end
PBRM1-ChIP-seq.4h.fq.gz 21709461 16388887 150bp single-end
PBRM1-ChIP-seq.8h.fq.gz 23910215 17955411 150bp single-end
BRD7-ChIP-seq.0h.fq.gz 18943682 11523462 150bp single-end
BRD7-ChIP-seq.4h.fq.gz 20586502 12973126 150bp single-end
BRD7-ChIP-seq.8h.fq.gz 16562604 10411121 150bp single-end

Antibodies

anti-H3K27ac, abcam, ab4729; anti-SS18, CST, 21792; anti-cJUN, abcam, ab31419; anti-BRD7, Proteintech, 51009-2-AP-50ul; anti-PBRM1, Bethyl, A301-591A

Peak calling parameters

Peaks were identified using MACS2 with the parameter setting (--broad

Data quality

We evaluated the data quality by track view

Software

Bowtie2 (version 2.2.5), IGV (version 2.3.97), MACS2 (version 2.1.1), Deeptools (version 2.2.3)

Flow Cytometry

Plots

Confirm that:

- [x] The axis labels state the marker and fluorochrome used (e.g. CD4-FITC).
- [x] The axis scales are clearly visible. Include numbers along axes only for bottom left plot of group (a 'group' is an analysis of identical markers).
- [x] All plots are contour plots with outliers or pseudocolor plots.
- [x] A numerical value for number of cells or percentage (with statistics) is provided.

Methodology

Sample preparation

Cells were dissociated into single cell using 0.25% trypsin-EDTA, washed once with PBS and collected by centrifugation.

Instrument

Accuri C6 flow cytometer

Software

Accuri C6 Plus;FlowJo

Cell population abundance

50,000 cells were counted per sample analyzed. The cell population abundance we chosed is based on literatures in the field.

Gating strategy

Cell debris were excluded by FSC-A/SSC-A plot , OG2-ESCs were positive control

- [x] Tick this box to confirm that a figure exemplifying the gating strategy is provided in the Supplementary Information.

Magnetic resonance imaging

Experimental design

Design type

NO

Design specifications

NO

Behavioral performance measures

NO

Acquisition

Imaging type(s)	NO
Field strength	NO
Sequence & imaging parameters	NO
Area of acquisition	NO

Diffusion MRI ☐ Used ☒ Not used

Preprocessing

Preprocessing software	NO
Normalization	NO
Normalization template	NO
Noise and artifact removal	NO
Volume censoring	NO

Statistical modeling & inference

Model type and settings	NO
Effect(s) tested	NO

Specify type of analysis: ☐ Whole brain ☐ ROI-based ☐ Both

Statistic type for inference (See Eklund et al. 2016)	NO
Correction	NO

Models & analysis

n/a	Involved in the study
☒	☐ Functional and/or effective connectivity
☒	☐ Graph analysis
☒	☐ Multivariate modeling or predictive analysis

Functional and/or effective connectivity	NO
Graph analysis	NO
Multivariate modeling and predictive analysis	NO
