# Supplementary material for: SS18 regulates pluripotent-somatic transition through phase separation
Source: Nat Commun. 2021 Jul 2;12:4090. doi: 10.1038/s41467-021-24373-5 (PMC8253816; doi:10.1038/s41467-021-24373-5)
Supplement: Supplementary file 4 — Description of Additional Supplementary Files [file 41467_2021_24373_MOESM4_ESM.pdf]

Description of Additional Supplementary Files

File Name: Supplementary Data 1
Description: qPCR Primers used in this study.

File Name: Supplementary Data 2
Description: shRNA used in this study.

File Name: Supplementary Data 3
Description: CRISPR/Cas9 screen data processed by MAGeCK algorithm.

File Name: Supplementary Data 4
Description: IP-MS data analyzed using MaxQuant version 1.6.0.1 search against Mouse Fasta database, with label free quantification and match between runs functions enabled.
